# A Case of Rare Adipocytic Tumor of the Tongue: Lipoblastoma-Like Tumor With RB1 Gene Deletion

**DOI:** 10.7759/cureus.94365

**Published:** 2025-10-11

**Authors:** Yoshihiko Sugita, Hiromasa Hasegawa, Rita R Roy, Katsumitsu Shimada, Takanaga Ochiai, Motohiko Nagayama, Hatsuhiko Maeda

**Affiliations:** 1 Department of Oral Pathology/Forensic Odontology, School of Dentistry, Aichi Gakuin University, Nagoya, JPN; 2 Hard Tissue Pathology Unit, Graduate School of Oral Medicine, Matsumoto Dental University, Shiojiri, JPN; 3 Department of Physiology, Matsumoto Dental University, Shiojiri, JPN; 4 Department of Clinical Pathophysiology, Matsumoto Dental University, Shiojiri, JPN; 5 Department of Oral Pathology, Division of Oral Pathogenesis and Disease Control, School of Dentistry, Asahi University, Mizuho, JPN

**Keywords:** cd34, differential diagnosis, lipoblastoma-like tumor, oral adipocytic tumors, plag1, rb1 deletion, retinoblastoma protein, spindle cell lipoma, tongue

## Abstract

Lipoblastoma-like tumors (LLTs) are benign adipocytic tumors characterized by an admixture of lipoblasts within the myxoid stroma, primarily affecting the vulvovaginal area in young patients. Rare cases have been reported in other locations, including the orofacial region. Here, we present a unique case of LLT of the tongue in a 62-year-old woman with a slowly developing painless nodule. The tumor showed no signs of recurrence five years after surgery. Histopathological examination revealed a myxo-fatty lobulated lesion with numerous uni- and multi-vacuolated lipoblasts and prominent chicken-wire capillaries. Immunohistochemistry showed positivity for S100, whereas the tumor was negative for cluster of differentiation 34 (CD34), pleomorphic adenoma gene 1 (PLAG1), B-cell lymphoma 2* *(BCL-2), mouse double minute 2 homolog (MDM2), cyclin-dependent kinase 4 (CDK4), and DNA damage-inducible transcript 3 (DDIT3). The retinoblastoma protein (pRB) was partially negative and exhibited mosaic patterns. Fluorescence in situ hybridization (FISH) analysis indicated a hemizygous deletion of the *RB1* locus in 36% of tumor cells, with no *MDM2* amplification detected. This case highlights the diagnostic challenges of differentiating LLTs from spindle cell lipomas (SCLs), particularly in the context of pRB deficiency and *RB1* deletion. This case exemplifies the molecular heterogeneity of neoplasms with lipoblastoma-like features.

## Introduction

A lipoblastoma-like tumor (LLT) is a benign adipocytic tumor characterized by an admixture of lipoblasts, myxoid stroma, and spindle cells, which is reminiscent of lipoblastoma, myxoid liposarcoma, or spindle cell lipoma (SCL). Originally, LLTs were considered lesions that primarily affected the vulvovaginal area in young patients [1,2]. However, rare instances of LLTs affecting the paratesticular region have been reported, indicating that LLTs are not limited to the vulvo-inguinal region in women [3]. Anatomical sites of LLTs have included the retroperitoneum, pelvis, shoulder, and forearm in both women and men [4,5]. Across reported series, lesions typically enlarge slowly, occasionally exceeding 20 cm [2,4,5]. LLTs generally behave in an almost entirely benign manner [4] but exhibit local recurrence in half of the cases with positive excision margins [2,5]. Exceptionally, one case of vulvar LLT that developed pulmonary metastasis was reported among 28 cases of LLTs [5].

Oral cavity involvement is exceedingly uncommon, with only three sporadic cases of the lip and tongue documented to date [6-8]. Two cases of LLT originating from the lip [6,7] did not exhibit the characteristic features of LLTs, such as spindle cells, myxoid stroma, and chicken-wire capillaries [1]. Nevertheless, the presence of lobules containing mature adipocytes and lipoblasts was consistent with LLTs. The other, involving the tongue, harbored an *IDH1* mutation, a hitherto undescribed molecular alteration [8]. However, no additional cases of LLT affecting the intraoral mucosa have been reported.

The principal differential diagnoses of LLTs include myxoid liposarcoma, lipoblastoma, and SCL [1,2]. Ancillary testing aids in discrimination. Myxoid liposarcoma harbors *FUS/EWSR1-DDIT3* fusions, and nuclear DNA damage-inducible transcript 3 (DDIT3) immunoreactivity is a highly sensitive and specific marker [9]. Lipoblastoma typically occurs in infants and children and often shows pleomorphic adenoma gene 1 (PLAG1) overexpression and *PLAG1*/*HMGA2* rearrangements, with frequent cluster of differentiation 34 (CD34) and desmin expression [10,11]. SCL commonly exhibits diffuse strong CD34 and B-cell lymphoma 2 (BCL-2) staining and shows the loss of retinoblastoma protein (pRB) expression with *13q14*/*RB1* alterations in a substantial subset [12-15]. In contrast, LLT generally lacks PLAG1 expression/rearrangement and may show the complete or mosaic reduction of pRB, leading some authors to suggest a relationship with the SCL family while emphasizing distinct morphological and site predilections [2,4,5,15].

We report a unique adipocytic tumor of the tongue that presented with characteristic findings of LLT but exhibited a mosaic pRB pattern and a hemizygous loss of the *RB1 *locus. This case highlights the diagnostic challenges at the LLT-SCL interface and underscores the need for a practical, stepwise approach that integrates morphology, immunohistochemistry, and targeted molecular assays for the diagnosis of oral adipocytic lesions. Accurate diagnosis is essential because it supports conservative management for benign entities, prevents unnecessary treatment of lesions that resemble malignant tumors, and requires careful follow-up given the scarce long-term prognostic data for rare tumors such as LLT.

## Case presentation

Clinical summary

**Figure 1:**
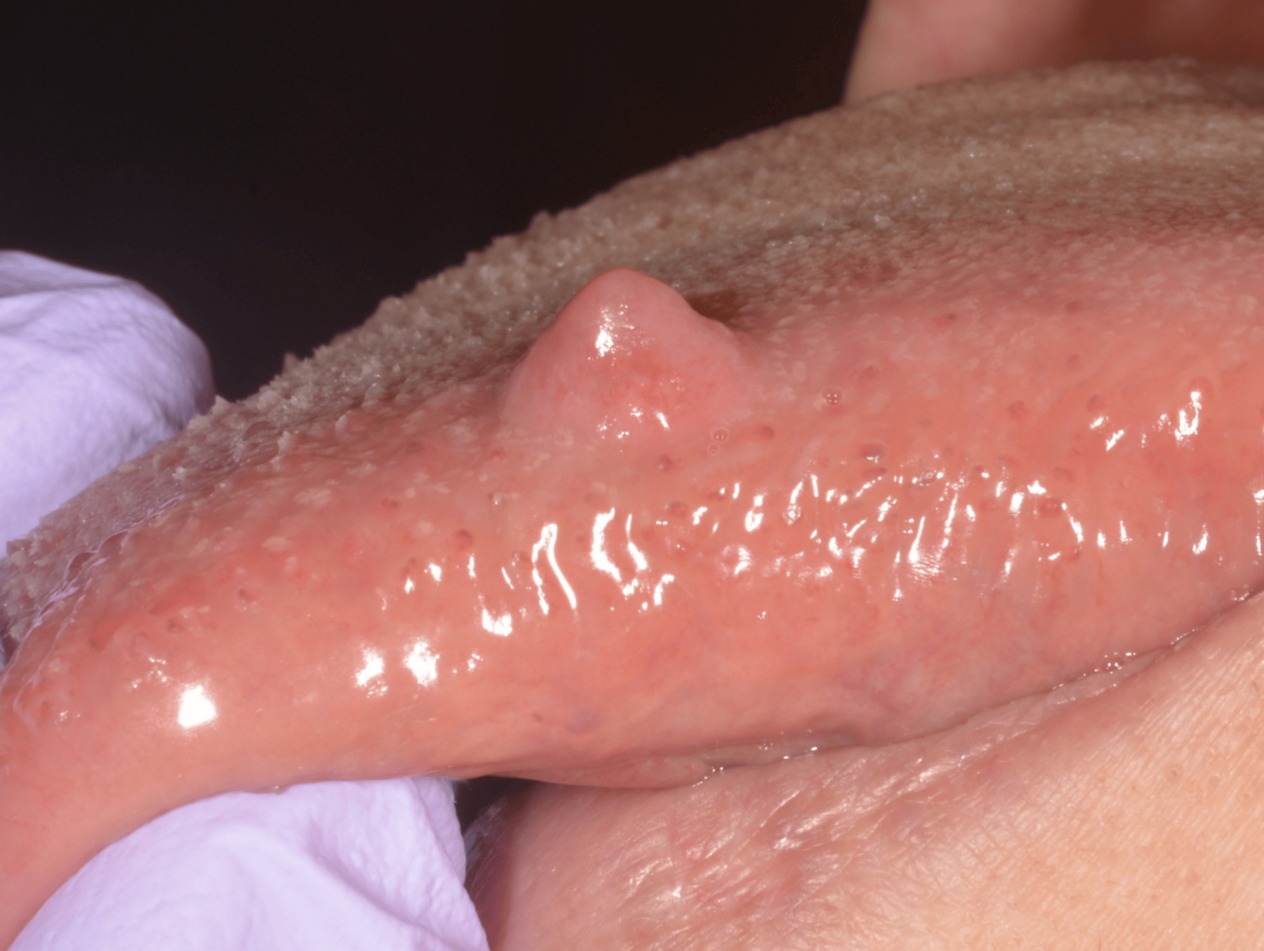
Clinical findings The intraoral manifestations present as an elevated lesion on the dorsal tongue

A 62-year-old woman presented with a painless nodule on her tongue covered by normal mucosa (Figure 1). The lesion developed slowly over seven years. The tumor was surgically resected under local anesthesia because it was clinically diagnosed as a lipoma. No sign of recurrence five years after surgery.

Pathological findings

**Figure 2:**
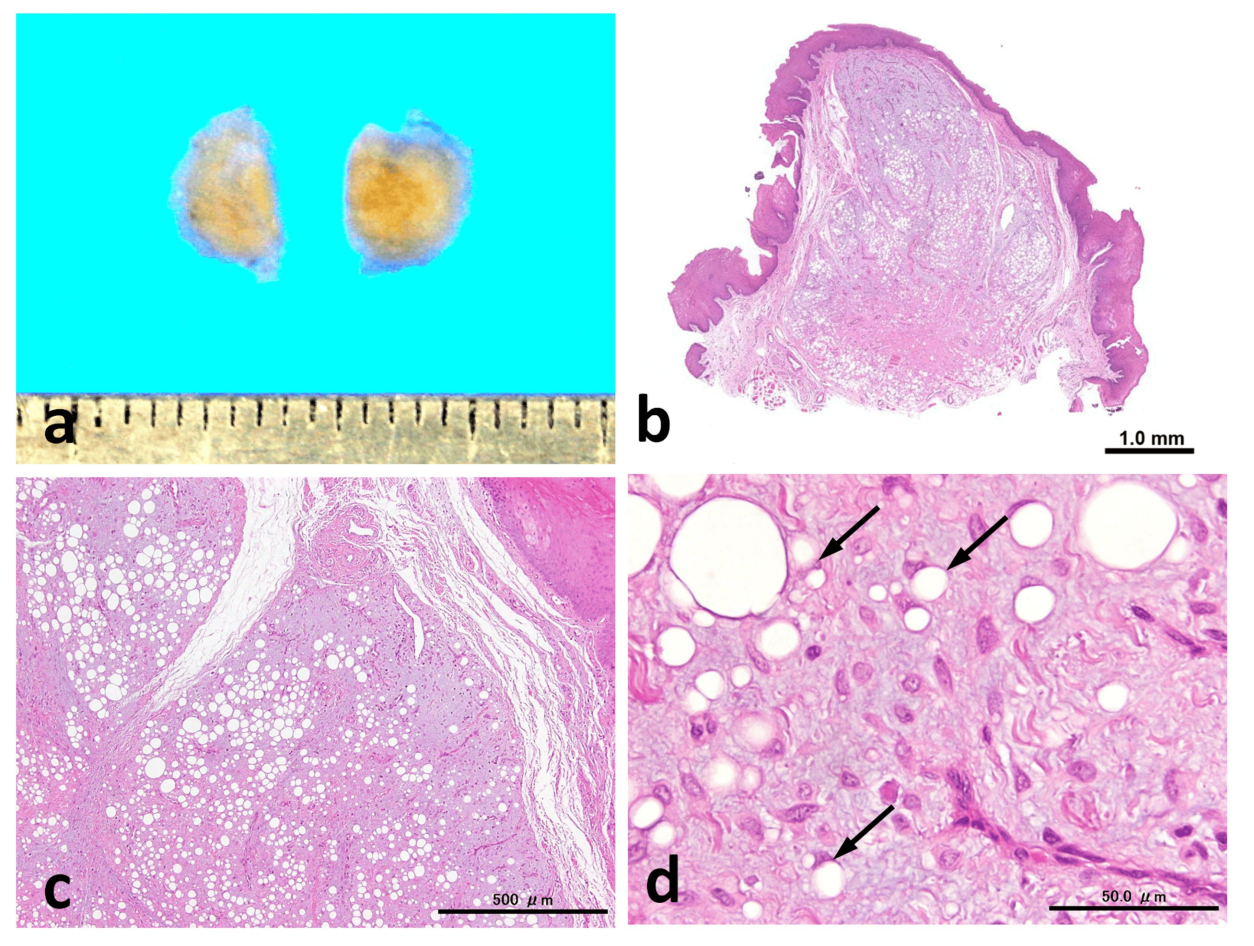
Histopathological findings The cut surface of the tumor appears yellow (a). Histologically, the tumor is unencapsulated (b) and composed of myxo-fatty lobules (c) made up of bland spindle cells, mature adipocytes, lipoblasts (arrows), and branching capillaries (d). Scale bars: 1.0 mm (b), 500 µm (c), and 50.0 µm (d)

The excised specimen measured 5 × 5.5 mm in size and had a yellowish cut surface with mucosal elevation (Figure 2a). Histologically, the lesion was relatively well-demarcated, unencapsulated, and lobulated with a grenz zone between the epithelium and the tumor. The lobules separated by fibrovascular septa predominantly consisted of myxoid stroma, accounting for approximately 60%-70% of the tumor, and scattered adipocytes. The adipocytes contained variably sized vacuoles resembling mature adipocytes and uni-, bi-, or multi-vacuolated lipoblasts. The myxoid stroma contained bland spindle cells without any atypical features. Numerous thin-walled branching capillaries with a chicken-wire appearance were observed within the myxoid stroma. Collagen fibers were scant, and no ropy collagen was observed (Figure 2b-2d).

**Figure 3:**
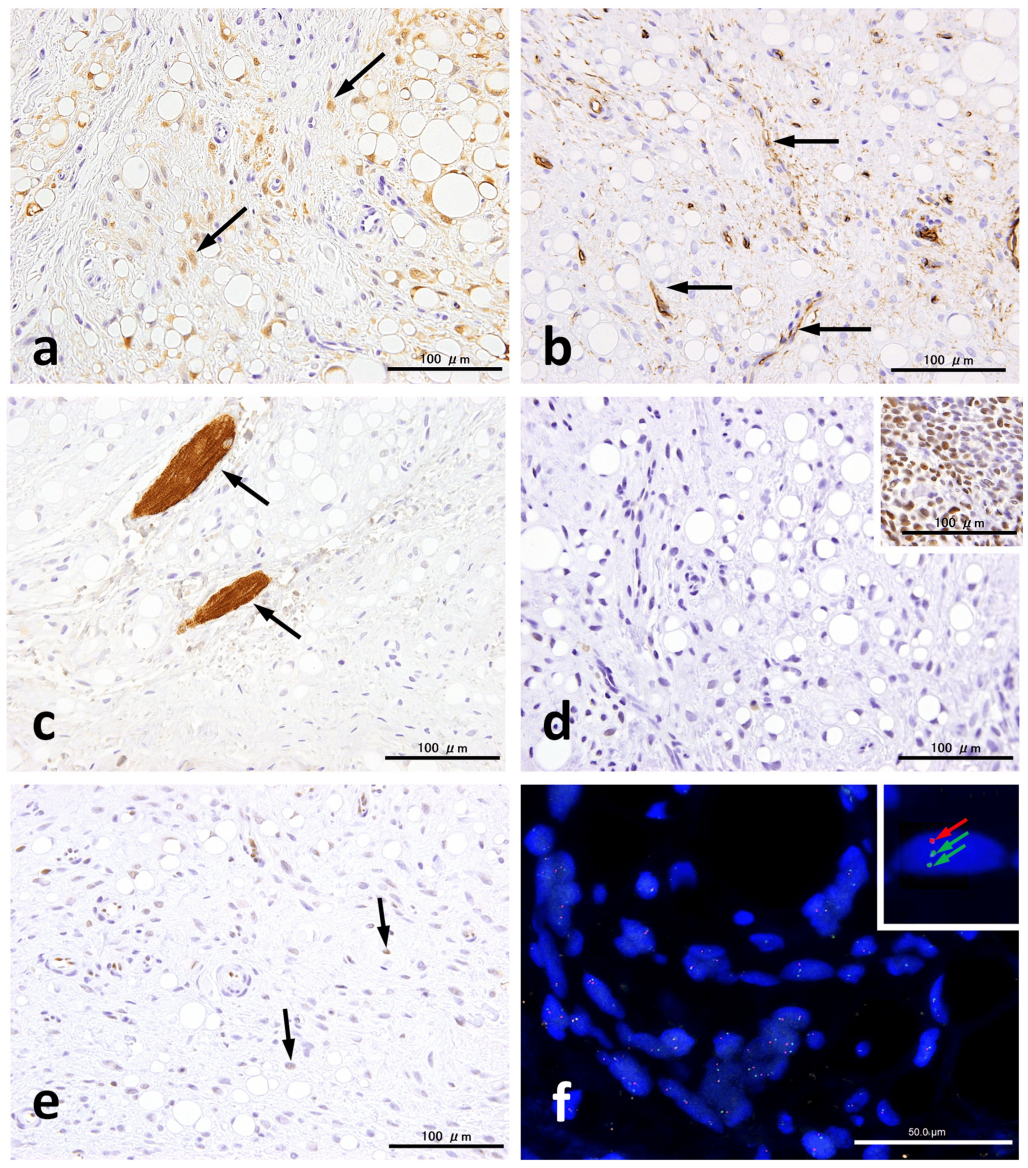
Immunohistochemistry and FISH analysis Immunohistochemistry shows spindle cells partially positive for S100 (a, arrows), while most are negative for CD34, which highlights the presence of capillaries (b). Desmin is completely absent in tumor cells but highlighted intermingled striated muscle fibers (c). PLAG1 expression is negative in contrast to the positive control case (inset) (d). pRB positivity is limited to spindle cells (arrows) and exhibits a mosaic pattern (e). *RB1* FISH demonstrates a hemizygous *RB1 *deletion, indicated by a single *RB1* signal (red arrow) and two centromere signals (green arrows) (inset). Scale bars: 50 µm (f) and 100 µm FISH, fluorescence in situ hybridization; CD34, cluster of differentiation 34; PLAG1, pleomorphic adenoma gene 1; pRB, retinoblastoma protein

Immunohistochemically, the variably sized adipocytic cells and some spindle cells were positive for S100 (polyclonal) (Figure 3a). CD34 (clone QBEnd/10) staining highlighted numerous small vessels but was mostly negative in tumor cells (Figure 3b). No desmin-positive tumor cells (clone D33) were identified (Figure 3c). However, desmin-positive striated muscle fibers were intermingled within the tumor. In contrast to the nuclear positivity for PLAG1 (clone 3B7) observed in a pleomorphic adenoma (Figure 3d), which served as a positive control, the tumor cells were completely negative. Some spindle cells were negative for pRB (clone G3-245), showing a mosaic staining pattern (Figure 3e). Additionally, the cells were negative for BCL-2 (clone 124), mouse double minute 2 homolog (MDM2) (clone IF2), cyclin-dependent kinase 4 (CDK4) (clone DCS-31), and DDIT3 (clone 9C8) (data not shown).

Fluorescence in situ hybridization (FISH) was performed using ZytoLight SPEC *RB1*/*13q12* and *MDM2*/*CEN 12* (ZytoVision GmbH, Bremerhaven, Germany) dual-color probes. The analysis revealed a hemizygous deletion characterized by an orange signal corresponding to the *RB1* gene and two green centromere signals, indicating the presence of two chromosome copies in 36% of the analyzed tumor cells (Figure 3f). No *MDM2* amplification was detected (data not shown).

## Discussion

The characteristic morphology of a lobulated myxo-fatty tumor affecting the tongue is similar to that of lipoblastoma. However, the diagnosis of LLT may be more appropriate given the patient’s age (62 years) compared to the age of lipoblastomas at diagnosis, which ranges from three months to 16 years, with 90% of cases diagnosed before 10 years [16]. Nevertheless, the morphological characteristics of this case included a complete set of LLTs, and the heterozygous loss of* RB1* should be carefully considered.

LLTs affect the external genitalia, particularly the vulva, but rarely other sites [3-5]. LLTs of the oral cavity are rare, with only three cases previously reported [6-8]. These showed a mixture of mature adipocytic cells separated by thin collagenous septa containing small vessels or striated muscles but lacking spindle cells and myxoid elements. However, the overall features of these cases, including CD34 negativity, were consistent with those of LLT.

The morphological features of LLT, rather than its clinical characteristics, suggest the need for a differential diagnosis, which includes myxoid liposarcoma, lipoblastoma, and SCL, as shown in Table 1 [1,2]. The *FUS/EWSR1-DDIT3* fusion characteristic of myxoid liposarcoma can be distinguished from LLT using DDIT3 immunohistochemistry, in which nuclear anti-DDIT3 immunoreactivity is a highly sensitive and specific marker of myxoid/round cell liposarcoma [9].

**Table 1:** Comparative features of lipoblastoma, lipoblastoma-like tumor, spindle cell lipoma, and myxoid liposarcoma *Rarely affects adults ^#^Chromosomes 7, 9, 11, and 17 pRB, retinoblastoma protein; CD34, cyclin-dependent kinase 4; PLAG1, pleomorphic adenoma gene 1; DDIT3, DNA damage-inducible transcript 3

Findings	Lipoblastoma	Lipoblastoma-like tumor	Spindle cell lipoma	Myxoid liposarcoma
Age	Children*	Adult	Adult	Adult
Lobulation	Present	Present	Absent	Present
Lipoblasts	Abundant	Abundant	Scant	Scant
Myxoid	Abundant	Abundant	Variable	Abundant
Capillary network	Abundant	Abundant	Scant	Abundant
CD34	Negative	Negative/focal	Positive	Negative
S100	Positive	Positive	Focal	Focal
PLAG1	Positive	Negative	Negative	Negative
Desmin	Positive	Negative	Negative	Negative
pRB loss	Negative	Mosaic	Positive	Negative
DDIT3	Negative	Negative	Negative	Positive
Gene alteration	PLAG1	Variable gains and losses^#^	*RB1* loss	FUS-DDIT3

Lipoblastomas typically occur in infants and children, and even in older children and adult patients, positivity for PLAG1, CD34, and desmin, along with *PLAG1* and *HMGA2* rearrangements, aids in diagnosis [10,11]. The histological findings in this case showed myxoid stroma, spindle cells, chicken-wire capillaries, and a mixture of mature adipocytes and lipoblasts, compatible with LLTs. Additionally, negative results for DDIT3, CD34, desmin, and PLAG1 support the diagnosis and help exclude myxoid liposarcoma and “adult” lipoblastoma [9-11].

Finally, differentiating between LLT and SCL is a significant issue in this case, as they share similar clinical characteristics except for their sites of occurrence [14,16]. Histologically, some SCLs with prominent lipoblasts closely resemble LLTs or lipoblastomas [17]. Additionally, desmin immunohistochemistry revealed that the tumor involved lingual muscle fibers, which may indicate the infiltrative nature of lipoblastoma rather than SCLs or LLTs [16].

From an immunohistochemical perspective, the absence of CD34 and BCL-2 expression in spindle cells suggests LLT, because SCLs typically exhibit diffuse and strong immunostaining for CD34 and BCL-2 [12,14]. Although pRB expression patterns in LLTs can vary, cases exhibiting complete loss or mosaic patterns are challenging to differentiate from SCL [4,18]. In this case, pRB demonstrated a mosaic pattern, which prompted us to perform *RB1 *FISH analysis. Contrary to our expectations, 36% of nuclei exhibited a hemizygous deletion of the *RB1* locus. This result raises uncertainty as to whether the case is an LLT.

The frequencies of *RB1* loss in SCLs vary, accounting for roughly 40%-60%, in contrast to the pRB deficiency observed in almost all cases [13]. Additionally, numerical and/or structural changes were found not only on chromosome 13 but also on chromosomes 1, 6, 11, and 12 [15]. Mirkovic and Fletcher reported that six cases of LLT of the vulva showed immunohistochemical loss of pRB but no expression of PLAG1, suggesting a relationship with the SCL tumor family [2]. However, molecular analyses were not performed in their study.

Another study showed that LLTs immunohistochemically exhibited a mosaic pRB pattern, and no evidence of *RB1* regional gain or loss was identified using FISH [18]. As reported by Gross et al., several chromosomal abnormalities were noted, including gains on chromosomes 7 and 9, losses at 17q, and copy-neutral loss of heterozygosity at 11p, in three of eight cases tested using a chromosomal microarray [4]. Chromosomal aberrations were not observed on chromosome 13. Additionally, individual cases of LLT harboring mutations in *MTOR*, *IDH1*, *PIK3CA*, *CSF1R*, and* CDKN2A/B*,* *on chromosomes 1, 2, 3, 5, and 9, respectively, have been reported [4,5,8]. Given that these molecular aberrations vary between SCLs and LLTs, both lesions are heterogeneous with respect to chromosomal abnormalities. The hemizygous *RB1* deletion in this case suggests an unusual variant of SCL that lacks CD34 and BCL-2. However, the morphological and immunohistochemical characteristics supported the diagnosis of LLT with *RB1* loss.

This case highlights the challenge of distinguishing between LLT and SCL when confronted with conflicting immunohistochemical and genetic data. As mentioned above, we cannot completely rule out the possibility that this case represents an atypical example of SCL. Except for the presence of a hemizygous *RB1* deletion, the morphological and immunohistochemical features were consistent with those of an LLT. Although LLT is not currently listed in the WHO classification of soft tissue tumors [19], likely due to its rarity and morphological overlap with established entities, the well-documented features of LLTs support the use of the term “lipoblastoma-like” for this diagnosis, as shown in the original report [1].

A limitation of this case report is the lack of a comprehensive molecular analysis, which hinders the provision of precise diagnostic information. Given the heterogeneity of the molecular characteristics of LLTs and SCLs, this case should be provisionally diagnosed based on the available morphological and immunohistochemical findings. Accumulating more cases involving extragenital areas is essential in further elucidating their nature and improving diagnostic accuracy.

## Conclusions

This case illustrates that an LLT of the tongue can display classic morphology with hemizygous *RB1* loss. Despite this genetic finding, the overall profile, DDIT3-negative, PLAG1-negative, and CD34/BCL-2-negative, supports an LLT over SCL. The clinical significance of *RB1* alterations in LLTs is unknown. Broader molecular profiling across sites and larger cohorts is needed to clarify the boundaries between LLT and SCL and refine the terminology and criteria for these overlapping entities.
